# TNFα and IL-1β influence the differentiation and migration of murine MSCs independently of the NF-κB pathway

**DOI:** 10.1186/scrt492

**Published:** 2014-08-27

**Authors:** Catherine B Sullivan, Ryan M Porter, Chris H Evans, Thomas Ritter, Georgina Shaw, Frank Barry, Josephine Mary Murphy

**Affiliations:** Regenerative Medicine Institute, National University of Ireland Galway, University Road, Galway, Ireland; Center for Advanced Orthopedic Studies, Beth Israel Deaconess Medical Center, Research North 115, 99 Brookline Ave, Boston, MA 02215 USA

## Abstract

**Introduction:**

Mesenchymal stem cells (MSCs) have the ability to repair and regenerate tissue, home to sites of inflammation, and evade the host immune system. As such, they represent an attractive therapy for the treatment of autoimmune inflammatory diseases. However, results from *in vivo* murine studies in inflammatory arthritis have been conflicting, and this may be due to the genetic background of the MSCs used. It is known that the inflammatory milieu may influence properties of MSCs and that, in the case of human bone marrow-derived MSCs, this may be mediated by the nuclear factor-kappa-B (NF-κB) pathway. We sought to determine whether pro-inflammatory cytokines altered the differentiation and migration capacity of murine MSCs from different mouse strains and whether this was mediated by NF-κB.

**Methods:**

The differentiation and migration of FVB and BALB/c MSCs were carried out in the presence of varying concentrations of tumor necrosis factor-alpha (TNFα) and interleukin (IL)-1β, and the NF-κB pathway was inhibited in one of two ways: either by transduction of MSCs with an adenoviral vector expressing a super-repressor of NF-κB or by the addition of curcumin to culture media.

**Results:**

Both BALB/c and FVB MSCs were sensitive to the effect of pro-inflammatory cytokines *in vitro*. TNFα and IL-1β suppressed BALB/c osteogenesis and adipogenesis and FVB osteogenesis. The migration of both cell types toward media containing fetal bovine serum was augmented by pre-stimulation with either cytokine. In neither cell type were the cytokine effects reversed by abrogation of the NF-κB pathway.

**Conclusions:**

These data show that murine MSCs from different genetic backgrounds may be influenced by an inflammatory milieu in a manner that is not mediated by NF-κB, as is the case for human MSCs. This is not mediated by NF-κB. These findings are important and should influence how *in vivo* trials of murine MSCs are interpreted and the future development of pre-clinical studies in inflammatory diseases.

**Electronic supplementary material:**

The online version of this article (doi:10.1186/scrt492) contains supplementary material, which is available to authorized users.

## Introduction

Mesenchymal stem cells (MSCs) have been isolated from several sites, including bone marrow and adipose tissue, and under appropriate stimuli can undergo osteogenesis, adipogenesis, and chondrogenesis [1]. MSCs can also modulate the responses of B and T cells to various immune responses *in vitro*. Proliferation of CD4^+^ and CD8^+^ T cells can be inhibited in a dose-dependent manner independently of major histocompatibility complex (MHC) matching with reduced expression of activation markers [2, 3]. Immune regulation by MSCs may be mediated through secondary effects on other cells such as decreased tumor necrosis factor alpha (TNFα) and increased interleukin-10 (IL-10) production from dendritic cells, decreased T helper 1 (Th1) and natural killer cell production of interferon-gamma (IFNγ), and the generation of antigen specific T regulatory cells [1, 4–8]. Mechanistically, this immune suppression may be mediated by transforming growth factor-beta, hepatocyte growth factor, IL-10, or prostaglandin-E_2_ production [2, 5, 9, 10]. In addition to these characteristics, MSCs are considered immune privileged cells as they have low MHC II expression and lack co-stimulatory molecules [11–13]. These features, in combination with their potential to repair and regenerate, make MSCs a potentially attractive option for the treatment of immune-mediated inflammatory conditions associated with tissue destruction.

One such example is in inflammatory arthritis where, despite some promising *in vitro* data [14], studies assessing the effect of MSCs in murine collagen-induced arthritis (CIA) have generated conflicting results, which may be partly attributed to differences in the number of cells used, the route of delivery, and timing of administration. Also of importance is consideration of the source of the MSCs used and the method of isolation; many studies have used marrow-derived MSCs while some are isolated from adipose tissue or indeed are cell lines derived from embryonic mesoderm. The differences in isolation and expansion techniques used may have an unknown impact on the ability of the MSCs to suppress inflammation *in vivo* [15–23]. Additionally, it has been demonstrated that both the genetic background of the MSCs and the local inflammatory milieu may impact on the ability of MSCs to modulate disease activity *in vitro* [19, 24].

Pro-inflammatory cytokines such as TNFα have significant effects on human MSCs; they can modulate proteins linked to immunosuppressive and signaling pathways such as manganese superoxide dismutase (SOD2), ribose-phosphate pyrophosphokinase 1, septin-9, and signal transducer and activator of transcription 1 (STAT1) [25]; enhance migration [26]; and inhibit chondrogenesis and osteogenesis [27, 28]. There is a negative relationship between the magnitude of synovial inflammation and the chondrogenic and clonal capacities of MSCs isolated from rheumatoid synovium, further supporting the hypothesis that the inflammatory milieu alters MSC characteristics [29]. Differentiation of murine MSCs is also altered by the inflammatory milieu. Decreased expression of osteoblast marker genes in TNFα transgenic mice and suppression of osteogenesis in C57BL/6 MSCs after treatment with TNFα or IL-1β have been described [30, 31]. Pro-inflammatory cytokines can also alter the effect of MSC proliferation *in vitro* and promote MSC-mediated tumor growth [20, 32].

The mechanism underlying these effects is poorly understood. Upregulation of ubiquitin ligases has been implicated as has β-catenin-mediated upregulation of Wnt signaling [32, 30] while both TNFα and IL-1 have major effects on mitogen-activated protein kinases (MAPKs) and phosphoinositide 3 kinase (PI3K) pathways, which have been shown to mediate migration and proliferation in chondrocytes and fibroblasts [33–35]. The transcription factor nuclear factor-kappa-B (NF-κB) plays a central role in coordinating the immune response in rheumatoid arthritis and CIA through transcription of cytokine genes, including TNFα and IL-1β, regulation of genes that influence cell migration as well as differentiation and proliferation, induction of adhesion molecules, increased vascular permeability, and recruitment of inflammatory cells [36]. The inhibition of chondrogenesis by IL-1β and TNFα in human MSCs is regulated by NF-κB [27] while activation of this pathway may stimulate osteogenesis in human adipose-derived MSCs [37]. Additionally, TNFα-mediated increases in MSC migration are reversed by inhibition of the NF-κB pathway [38]. As well as the effects on differentiation and migration, there are data to suggest that stressors such as TNFα and hypoxia can upregulate the production of vascular endothelial growth factor from human MSCs in an NF-κB-dependent manner [39]. There is less information on the role of NF-κB on murine MSCs, although there are data showing that migration of C57BL/6 MSCs toward epithelial cells infected with *Helicobacter pylori* is mediated by NF-κB-driven upregulation of TNFα [40]. Additionally, chemical inhibition of the NF-κB pathway with curcumin can facilitate chondrogenesis in canine MSCs [41].

Taking these data into consideration and having demonstrated that the genetic background of MSCs may affect their ability to modulate inflammatory responses [19], we sought to determine whether mouse MSCs from varying backgrounds responded differently to pro-inflammatory cytokines in terms of their fundamental properties of differentiation and migration. Given the significant role of NF-κB in mediating the effect of inflammation on human MSCs, we also sought to determine whether this pathway is relevant in murine models. BALB/c and FVB MSCs represent two allogeneic strains of MSCs with a variable degree of genetic mismatch relative to the DBA/1 strain used in CIA, and we hypothesized that co-culture with either TNFα or IL-1β would influence the differentiation and migratory capacity of these MSCs to varying degrees. Given the significant role of NF-κB in mediating the effect of inflammation on human MSCs, we also sought to determine whether this pathway is relevant in murine models.

## Materials and methods

### MSC isolation

All animal work was carried out with approval from the National University of Ireland Galway Animal Care Research Ethics Committee. Bone marrow MSCs were isolated from 8- to 10-week-old BALB/c (Harlan Laboratories, Indianapolis, IN, USA) and FVB.Cg-Tg(GFPU)5Nagy/J (FVB-GFP) (The Jackson Laboratory, Bar Harbor, ME, USA) as previously described [19]. Briefly, marrow mononuclear cells obtained from long bones were plated in cold complete isolation medium (CIM): Roswell Park Memorial Institute (RPMI) 1640, 9% horse serum (HS), 9% fetal bovine serum (FBS), 1% penicillin/streptomycin (P/S), 1% L-glutamine. After 24 hours, cultures were washed with phosphate-buffered saline (PBS) and fresh CIM was added. Medium was changed every 3 to 4 days, and large colonies were seen after approximately 4 weeks in passage 0 (P0). Cells were replated in a new T175 flask in CIM for 14 days. Thereafter, cells were replated at 500 cells per cm^2^ and culture expanded in complete expansion medium (CEM): Iscove’s modified Eagle’s medium, 9% HS, 9% FBS, 1% P/S, 1% L-glutamine.

### Differentiation of MSCs

MSCs were stimulated to undergo adipogenesis, osteogenesis, and chondrogenesis as previously described [19]. Briefly, CEM was used as the basal medium for both adipogenesis and chondrogenesis. Adipogenic medium contained supplemental 1 mM dexamethasone, 100 nM indomethacin, insulin, rabbit serum, and high-glucose Dulbecco’s modified Eagle’s medium. Adipogenesis was confirmed with Oil Red O stain which was further extracted with isopropanol for determination of absorbance at 490 nm to allow comparison between culture conditions. For osteogenesis, MSCs were cultured with 20 mM β-glycerophosphate, 50 μM ascorbic acid 2-phosphate, and 100 nM dexamethasone. Fast Violet staining for alkaline phosphatase and alizarin red staining for calcium were carried out in the same wells. Calcium deposition was quantified as previously described [42] with a StanBio Calcium Liquicolour Kit (Stanbio Laboratory, Boerne, TX, USA). Where indicated, 10 or 25 μM curcumin was added to differentiation assays.

### Viral transduction

Optimization of viral transduction was carried out by using type V adenovirus expressing green fluorescent protein (AdGFP) supplied by Thomas Ritter (National University of Ireland Galway). Cells were plated at 5 × 10^4^ cells per well in a 12-well plate in CEM and allowed to adhere for 24 hours, medium was removed, and 300 μL of CEM containing AdGFP was added to appropriate wells. In initial experiments, virus was added at 1,000, 500, or 100 infectious viral particles per cell (multiplicity of infection, or MOI). Control wells had 300 μL of CEM alone added, and cultures were centrifuged at 2000 *g* at 37°C for 90 minutes. After this, medium was replaced with 2 mL of CEM. Cells were incubated for 48 hours at 37°C and 5% CO_2_ before washing with PBS and trypsinization. Cells were transferred to 1.5-mL tubes, pelleted, resuspended in PBS, and transferred to a round-bottomed 96-well plate. Four microliters of 7-aminoactinomycin D (7-AAD) was added to appropriate wells and incubated on ice for 15 minutes, allowing cells with compromised cell membranes to take up the dye. Cells were fixed in 4% paraformaldehyde for 20 minutes and resuspended in 200 μL of PBS. GFP expression was analyzed on a Guava Flow Cytometer with eXpress Plus™ software (EMD Millipore, Billerica, MA, USA) and expressed as percentage GFP expression in cell population. Based on results, further viral transduction was carried out at MOI 500.

Type V adenovirus expressing the super-repressor inhibitor of NF-κB (srIκB) [27] and MSCs were transduced at an MOI of 500. Expression of srIκB by virally transduced cells was confirmed by Western blot. Briefly, transduced cells were lysed, and total protein concentration was determined by using the bicinchoninic acid protein assay (Thermo Fisher Scientific, Waltham, MA, USA) in accordance with the instructions of the manufacturer. Equal amounts (60 μg) of protein were resolved by SDS-PAGE on a 12% polyacrylamide gel and transferred to a polyvinylidene difluoride membrane. Rabbit antibodies against human IκBα and β-actin (Santa Cruz Biotechnology, Santa Cruz, CA, USA) were used for immunodetection with a horseradish peroxidase-conjugated goat anti-rabbit IgG (Chemicon, Temecula, CA, USA). Bands were visualized by enhanced chemiluminescence by using a FlourChem™ image station (Alpha Innotech, San Leandro, CA, USA).

For assessment of NF-κB activity, MSCs were seeded in 96-well plates and after 24 hours were transduced with AdsrIκB or Adnull prior to transduction with either an NF-κB- or a cytomegalovirus (CMV)-driven luciferase reporter (AdNF-κB-Luc or AdCMV-Luc) [27]. Medium was changed every 72 hours; after 6 days, cells were stimulated with 5 ng/mL murine (m) TNFα, 10 ng/mL mIL-1β, or 100 ng/mL mIL-6 (Peprotech, London, UK). Four hours after stimulation, cell layers were collected in 20 μL of lysis buffer (Promega, Southampton, UK) and luciferase activity was determined by using the Dual-Glo™ Luciferase Assay System (Promega). Activity from AdNF-κB-Luc groups was normalized to that from matched AdCMV-Luc controls.

### MSC migration assays

*In vitro* migration assays were carried out by using 24-well Corning Transwell Inserts (Sigma-Aldrich, St. Louis, MO, USA) with a surface growth area of 0.33 cm^2^ and 8-μm pores. Inserts were pre-wetted with serum-free media, and 6 × 10^3^ MSCs in 200 μL serum-free medium was seeded onto the upper surface of the transwell. Either untransduced MSCs or MSCs expressing srIκB were used. Where necessary, cells were pre-stimulated for 24 hours with 50 ng/mL TNFα or IL-1β (Peprotech) in CEM. The seeded transwell was placed in a well with 600 μL of the appropriate medium, containing curcumin where indicated, and incubated at 37°C and 5% CO_2_. After 18 hours of incubation, cells remaining on the upper surface of the membrane were removed gently with a wetted cotton swab. The cells which had migrated to the lower surface were fixed in ice-cold methanol. Nuclei were stained for 15 minutes in Harris hematoxylin, and excess stain was removed with tap water. The membranes were dried, removed from the inserts with a scalpel, and mounted on glass slides by using oil emulsion mounting medium. Images were taken by using an Olympus IX81 upright microscope, and migrating cells were counted by using CellIP software (Olympus, Tokyo, Japan).

### Statistics

Statistical analysis was performed using StatsDirect® software (StatsDirect Ltd., Altrincham, UK). The Shapiro-Wilks test was used to confirm a normal distribution of data. Two-way analysis of variance with *post hoc* Tukey analysis was used for comparison between groups. Results are presented as the mean ± standard error of the mean.

## Results

### MSC isolation and viral transduction

**Figure 1:**
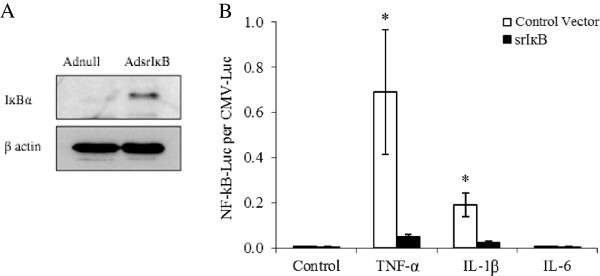
**Overexpression and functional activity of super-repressor inhibitor of nuclear factor-kappa-B (srIκB) in BALB/c mesenchymal stem cells (MSCs).** Western blotting demonstrated that BALB/c MSCs transduced with AdsrIκB at multiplicity of infection (MOI) 1,000 expressed higher levels of IκB than cells transduced with adnull virus **(A)**. MSCs were transduced with nuclear factor-kappa-B (NF-κB)-driven luciferase and either AdsrIκB or AdGFP at MOI 500. After stimulation with tumor necrosis factor-alpha (TNFα) or interleukin-1 (IL-1), luciferase activity was demonstrated in MSCs transduced with control virus, confirming upregulation of the NF-κB pathway. Conversely, MSCs transduced with AdsrIκB showed significantly less luciferase activity, indicating successful inhibition of the pathway. The NF-κB pathway was not stimulated by IL-6 in either group **(B)**. Cytokines were used at a concentration of 100 ng/mL. Data are presented as mean ± standard error of the mean of triplicate experiments. *P <0.05 (two-way analysis of variance).

Cells in P2 were homogenous in appearance with a fibroblastic morphology and were over 95% positive for CD29. Both cell types were negative for the hematopoietic markers CD45 and CD31 and the granulocyte marker CD34. Both cells types were successfully stimulated to undergo adipogenesis, osteogenesis, and chondrogenesis (not shown) in response to appropriate stimuli [19]. This method of MSC isolation from mouse femurs has been reported elsewhere [43]. After P2, 7-AAD staining was used to assess cell death, and 95% viability in both FVB and BALB/c cells was consistently demonstrated. Cells for the experiments described here were used at P3 to P6.BALB/c and FVB MSCs were transduced with AdsrIκB by using high-speed centrifugation over 90 minutes with negligible cell death. Western blotting confirmed overexpression of IκBα in transduced cells (Figure 1A) while functional assays confirmed a suppression of NF-κB-driven TNFα and IL-1β production compared with cells transduced with control vectors (Figure 1B).

### Suppression of osteogenesis and adipogenesis by pro-inflammatory cytokines

**Figure 2:**
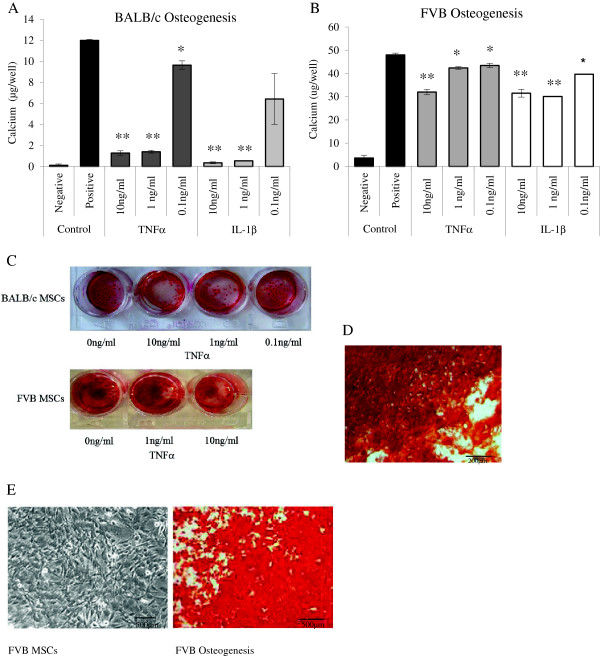
**Effect of inflammatory cytokines on osteogenesis of mouse mesenchymal stem cells (MSCs)** ***in vitro*** **.** Osteogenesis was suppressed in a dose-dependent manner by both tumor necrosis factor-alpha (TNFα) and interleukin-1-beta (IL-1β). This was statistically significant in **(A)** BALB/c and **(B)** FVB MSCs compared with MSCs differentiated in the absence of pro-inflammatory cytokines. MSCs cultured in complete expansion medium only did not undergo differentiation (negative control). The dose-dependent inhibition of osteogenesis in FVB and BALB/cMSCs was demonstrated by alizarin red staining **(C)**, and a representative micrograph of alizarin red staining of BALB/c MSCs is also shown **(D)**. FVB MSCs stimulated to undergo osteogenesis and stained with alizarin red and control FVB MSCs are shown **(E)**. Data shown are mean calcium per well ± standard error of the mean of triplicate experiments. *P <0.05, **P <0.001 (two-way analysis of variance).

**Figure 3:**
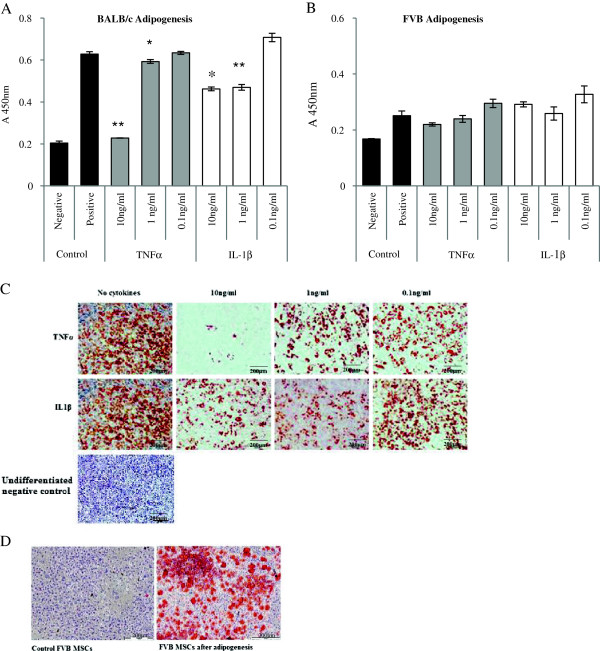
**Effect of inflammatory cytokines on adipogenesis of mouse mesenchymal stem cells (MSCs)** ***in vitro*** **.** Adipogenesis was suppressed by tumor necrosis factor-alpha (TNFα) and interleukin-1-beta (IL-1β) in a dose-dependent manner in **(A)** BALB/c MSCs compared with positive control cultures differentiated in the absence of pro-inflammatory cytokines. Negative controls represent MSCs cultured in complete expansion medium only. A similar trend was seen in **(B)** FVB MSCs but did not reach statistical significance. The effect of pro-inflammatory cytokines on BALB/c adipogenesis was also demonstrated by Oil Red O staining **(C)**. A representative image of Oil Red O staining of FVB cells cultured in adipogenic medium is shown **(D)**. Data shown are mean absorbance of dissolved Oil Red O ± standard error of the mean of triplicate experiments. *P <0.05, **P <0.001 (two-way analysis of variance).

Both BALB/c and FVB MSCs were stimulated to undergo osteogenesis. Representative images of alizarin red staining of FVB and BALB/c MSCs are shown in Figure 2D,E. FVB MSCs had a greater osteogenic potential, with almost 50 μg of calcium deposited per well compared with 12 μg in comparable BALB/c cultures. Osteogenesis as measured by calcium deposition was suppressed in both cell types by TNFα and IL-1β. The absolute decrease in calcium suppression was similar across both cell types (Table 1). In terms of percentage decrease in calcium production, the effect was most pronounced in BALB/c MSCs and occurred in a dose-dependent manner, with an 86% reduction in calcium deposition with 1 to 10 ng TNFα, a 17.5% reduction with 0.1 ng/mL TNFα, an almost 97% reduction with 1 to 10 ng/mL IL-1β, and a 50% reduction with 0.1 ng/mL of IL-1β (Figure 2A). This dose-dependent suppression was also seen in FVB MSCs but was less pronounced, with the maximum reduction of 37.5% seen after co-culture with 1 to 10 ng/mL IL-1β and 16.6% with 0.1 ng of IL-1β. Co-culture with TNFα at concentrations of 10, 1, and 0.1 ng/mL resulted in 35%, 14.6%, and 12.5% decreases in calcium deposition, respectively (Figure 2B). However, despite the lower percentage decrease in osteogenesis in FVB cells, it is worth noting that the decrease in calcium deposition when FVB MSCs were cultured with 10 ng/mL of either cytokine or with 1 ng/mL of IL-1β is more than the total calcium deposition of control BALB/c MSCs. The suppression of BALB/c osteogenesis by TNFα was confirmed by a decrease in mineral deposition by alizarin red staining (Figure 2C). Magnified images of alizarin red stained BALB/c and FVB MSCs stimulated to undergo osteogenesis are also shown (Figure 2D and E).Adipogenesis was induced in both BALB/c and FVB MSCs, with BALB/c demonstrating a higher adipogenic potential with an Oil Red O value of 0.6 at 450 nm. Adipogenesis in BALB/c was reduced by 66% when 10 ng/mL TNFα was added to the culture medium. A lower concentration of TNFα suppressed adipogenesis only marginally, 4.8% reduction with 1 ng/mL TNFα, while IL-1β at 10 or 1 ng/mL reduced adipogenesis by approximately 25%. The addition of either cytokine at 0.1 ng/mL had no significant effect on BALB/c adipogenesis (Figure 3A). Representative images of the suppression of BALB/c MSC adipogenesis by TNFα and IL-1β are shown in Figure 3C. FVB MSCs had less adipogenic potential that BALB/c MSCs, and there was no suppression of this differentiation pathway by either pro-inflammatory cytokine at any dose (Figure 3B,D).

**Table 1:** **Absolute decrease in calcium per well of mesenchymal stem cells undergoing osteogenesis in the presence of pro-inflammatory cytokines**

	Decrease in absolute calcium per well, mean μg (SD)		***P***value
	BALB/c	FVB	
10 ng/mL TNFα	10.72 (0.55)	16.71 (1.47)	0.03^a^
1 ng/mL TNFα	10.6 (0.41)	5.4 (2.61)	0.09
0.1 ng/mL TNFα	2.35 (0.54)	4.25 (3.45)	0.36
10 ng/mL IL-1β	11.64 (0.03)	16.52 (4.97)	0.23
1 ng/mL IL-1β	11.45 (0.20)	17.88 (1.87)	0.03^a^
0.1 ng/mL IL-1β	5.57 (4.36)	8.16 (4.44)	0.65

The absolute decrease in calcium per well of FVB and BALB/c cells stimulated to undergo osteogenesis in the presence of pro-inflammatory cytokines. A decrease in calcium production was demonstrated in all wells with response by FVB cells statistically greater than that for BALB/c preparations in two culture conditions. ^a^ *P* <0.05. IL-1β, interleukin-1-beta; SD, standard deviation; TNFα, tumor necrosis factor-alpha.

### Cytokine mediated suppression of differentiation was not mediated by the NF-κB pathway

**Figure 4:**
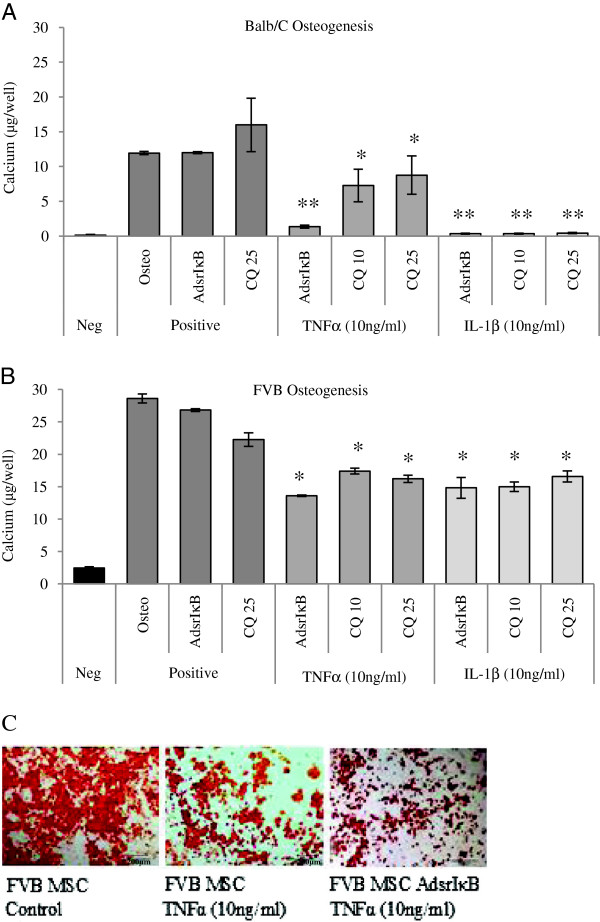
**Cytokine-mediated suppression of osteogenesis was not reversed by modulation of the nuclear factor-kappa-B (NF-κB) pathway.** Negative controls consisted of mesenchymal stem cells (MSCs) cultured in complete expansion medium, and positive controls represent MSCs stimulated to undergo osteogenesis, MSCs transduced with a super-repressor inhibitor of NF-κB (AdsrIκB) before differentiation, and MSCs differentiated in the presence of curcumin at a concentration of 25 μM (CQ 25). Osteogenesis was suppressed in **(A)** BALB/c and **(B)** FVB MSCs by 10 ng/mL tumor necrosis factor-alpha (TNFα) and interleukin-1-beta (IL-1β) compared with controls and was not rescued by MSC transduction with AdsrIκB or by the addition of curcumin at 10 μM (CQ 10) or 25 μM. The suppression of osteogenesis of BALB/c MSCs, irrespective of transduction with AdsrIκB, was also demonstrated by alizarin red staining **(C)**. Data shown are mean calcium per well ± standard error of the mean of triplicate experiments. *P <0.05, **P <0.001 (two-way analysis of variance).

**Figure 5:**
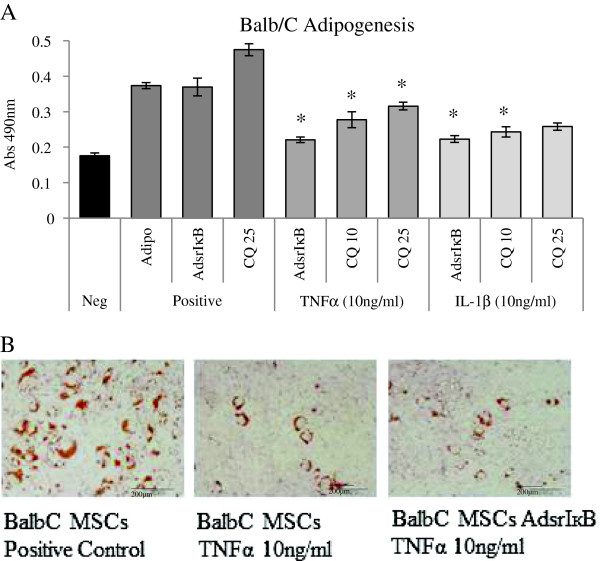
**Tumor necrosis factor-alpha (TNFα)-mediated suppression of adipogenesis was not reversed by modulation of the nuclear factor-kappa-B (NF-κB) pathway.** Negative controls consisted of mesenchymal stem cells (MSCs) cultured in complete expansion medium. Positive controls represent MSCs stimulated to undergo adipogenesis, MSCs transduced with AdsrIκB before differentiation (AdsrIκB), and MSCs differentiated in the presence of curcumin at a concentration of 25 μM (CQ 25). The suppression of adipogenesis by TNFα was statistically significant in **(A)** BALB/c MSCs compared with controls. Transduction of MSCs with AdsrIκB or addition of curcumin at 10 μM or 25 μM to culture medium (CQ 10, CQ 25) had no effect on differentiation and did not reverse the effect of TNFα. The reduction of adipogenesis in BALB/c MSCs by TNFα and the lack of effect of inhibition of the NF-κB pathway were also demonstrated by Oil Red O staining **(B)**. Data shown are mean absorbance of extracted Oil Red O ± standard error of the mean of triplicate experiments. *P <0.05 (two-way analysis of variance).

Differentiation studies were carried out with MSCs expressing AdsrIκB or with non-transduced MSCs in the presence of curcumin. The suppression of osteogenesis in BALB/c and FVB MSCs by 10 ng/mL of either pro-inflammatory cytokine was not rescued by inhibition of the NF-κB pathway by either of these methods (Figure 4A, B). Osteogenesis remained suppressed in terms of calcium deposition and mineralization (Figure 4C). Similarly, the suppression of adipogenesis by TNFα and IL-1β in BALB/c MSCs was not affected by interference of the NF-κB pathway as demonstrated by measurement of Oil Red O absorption (Figure 5A) and staining (Figure 5B).

### Pro-inflammatory cytokines enhanced migratory capacity of MSCs *in vitro*independently of the NF-κB pathway

**Figure 6:**
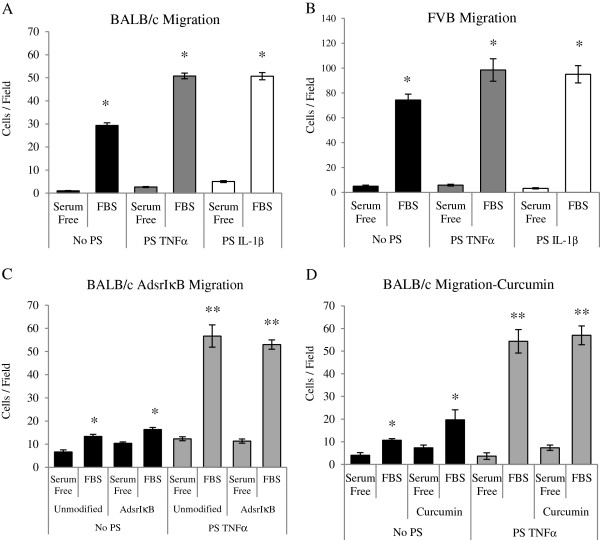
**Pre-stimulation (PS) with pro-inflammatory cytokines increased migration of murine mesenchymal stem cells (MSCs) toward fetal bovine serum (FBS)** ***in vitro*** **independently of the nuclear factor-kappa-B (NF-κB) pathway.** In BALB/c **(A)** and FVB **(B)** MSCs, a significant increase in migration toward 10% FBS was demonstrated compared with serum-free medium controls. PS of BALB/c and FVB MSCs with 50 ng/mL of tumor necrosis factor-alpha (TNFα) or interleukin-1-beta (IL-1β) for 24 hours increased migration of MSCs toward FBS medium compared with non-PS MSCs. Transduction of MSCs with AdsrIκB did not reverse the effect of pre-stimulation **(C)**. Similarly, addition of 25 μM curcumin did not reverse the effect of pre-stimulation with TNFα **(D)**. Data shown are the mean ± standard error of the mean of triplicate experiments. *P <0.05, **P <0.001 (two-way analysis of variance).

Both BALB/c and FVB MSCs migrated preferentially toward FBS-containing medium compared with serum-free medium (Figure 6A,B). When pre-stimulated with either TNFα or IL-1β, migration toward medium supplemented with FBS was significantly greater when compared with non-pre-stimulated control migration toward FBS (Figure 6A,B). The effect of cytokine pre-stimulation on migration was greatest in BALB/c MSCs and was not reversed by suppression of the NF-κB pathway either by viral transduction with AdsrIκB (Figure 6C) or by the addition of curcumin to the cultures (Figure 6D). Similarly, inhibition of the NF-κB pathway had no effect on the augmentation of FVB migration by pro-inflammatory cytokines (Additional file 1).

## Discussion

The regenerative capacity of MSCs holds great promise in the development of treatments for autoimmune inflammatory disorders characterized by tissue destruction. We have shown that the genetic background may influence the ability of MSCs to suppress inflammation in an allogeneic host: *in vitro* MSCs from various genetic backgrounds could suppress secretion of TNFα by activated DBA/1 T cells, but only syngeneic and partially mismatched MSCs suppressed IFNγ production [19]. Additionally, when comparing the overall effect of MSCs on the ratio of Th1 to Th2 cytokine production, fully allogeneic BALB/c MSCs were shown to have the least immunosuppressive effect. *In vivo* in a CIA model, allogeneic MSCs exacerbated disease progression and this was also evident biochemically with higher levels of IL-17 and IL-1β detectable in the sera of these mice [19]. A recent paper using allogeneic MSCs has demonstrated that MSCs may actually perpetuate inflammation through alterations in T-cell profiles and upregulation of Th-17 [44]. When the outcomes in these studies are considered, so too must be the potent pro-inflammatory milieu of CIA. CIA is predominantly a Th1-driven disease with Th2 cytokines present during remission. While TNFα and IL-1β are key mediators of inflammation, the role for IFNγ in CIA is complex with a peak in early disease and evidence of a disease-limiting role in late disease, decreasing IL-17 production and osteoclast precursors while increasing the activity of T regulatory cells [45–49]. Furthermore, data in antibody-induced arthritis in rats and spontaneous severe erosive arthritis in mice suggest that abrogation of the inflammatory milieu with a protease inhibitor may facilitate the immunosuppressive properties of MSCs in delivering a clinically apparent amelioration of disease [24].

Both TNFα and IL-1β have been shown to affect the characteristics of MSCs, and several pathways have been implicated in this. In particular, the NF-κB pathway modulates the differentiation and migration of human MSCs [27, 37, 50], and there are some limited data on the role of NF-κB in the migration of murine MSCs [40]. Here, we have looked at the effects of TNFα and IL-1β, key cytokines in RA and CIA, on the ability of murine MSCs to differentiate and migrate *in vitro*. It has been clearly demonstrated that both BALB/c and FVB MSCs have less osteogenic and adipogenic potential in the presence of pro-inflammatory cytokines. FVB MSCs were shown to have a greater osteogenic potential than BALB/c MSCs when cultured under the same conditions; however, both had a similar absolute reduction in calcium deposition when co-cultured with inflammatory cytokines. In the case of adipogenesis, BALB/c MSCs consistently demonstrated a higher adipogenic capacity than FVB MSCs and were more sensitive to cytokine exposure, with an almost threefold reduction in lipid vacuole formation when incubated with higher doses of TNFα. Pro-inflammatory cytokines had no significant effect on FVB MSC adipogenesis, nor did they influence the osteogenesis or adipogenesis of MSCs isolated from DBA/1 mice (results not shown).

Both BALB/c and FVB MSCs migrated preferentially toward medium containing FBS *in vitro*, compared with serum-free medium, with a higher migratory capacity consistently demonstrated in FVB cultures. This migration was augmented by pre-stimulation with TNFα and IL-1β at relatively high doses in both cell types. The magnitude of this effect was similar in both cell types, with an approximately 1.5-fold increase in the number of cells migrating.

Owing to the central role of NF-κB in the perpetuation of immune activation in arthritis and its importance in the response of human MSCs to inflammation, it was hypothesized that this pathway was likely to be involved in the response of murine MSCs to pro-inflammatory cytokines. Curcumin is derived from turmeric and has been shown to inhibit NF-κB as well as downregulating cyclooxygenase-2, nitric oxide synthase, and matrix metalloproteinase-9 [51]. It has also been reported that curcumin can promote osteogenesis and inhibit adipogenesis in rat MSCs, although the mechanism for this is unclear [52]. A pathway of particular interest is the p38 MAPK pathway, and it has been demonstrated that alterations in differentiation potential of human MSCs by modification of the actin cytoskeleton are associated with changes in the levels of phosphorylated p38 MAPK [53]. As well as a possible role in actin binding, p38 MAPK activity may affect bone morphogenetic protein (BMP)-regulated osteogenesis [54, 55]. Indeed, addition of curcumin may mediate some of its effect through the p38 MAPK pathway. The use of curcumin has been demonstrated to stimulate this pathway in some situations [56] whereas in others it may act through p38 inhibition [57]. One such study demonstrated that curcumin inhibited both NF-κB and MAPK pathways in TNFα-treated HaCaT cells, suggesting that the local inflammatory environment may affect the mode of action [58]. However, successful disruption of the NF-κB pathway with the super-repressor IκB was also insufficient to rescue either the differentiation or migratory capacity of either BALB/c or FVB MSCs in this study, confirming that this pathway is unlikely to be responsible for the effects seen.

Recently published *in vivo* data demonstrate that the inflammatory milieu interferes with the ability of MSCs to suppress the immune system and that this may be rescued by the use of protease inhibitors [24]. Here, we have demonstrated that pro-inflammatory cytokines found in inflammatory arthritis have the ability to affect some of the fundamental characteristics of MSCs, differentiation and migration, and furthermore that this effect is not consistent across genetic backgrounds. In contrast to the data on human MSCs, this phenomenon is not mediated by the NF-κB pathway. In light of this, other pathways such as MAPK and P13K will need to be looked at.

Interestingly, in contrast to the response to TNFα or IL-1β, stimulation of MSCs with IL-6 did not activate the NF-κB pathway in mouse MSCs as demonstrated with the NF-κB-driven luciferase. It has been suggested that IL-6 can increase migration of human MSCs *in vitro* and has important effects on rat MSC proliferation, migration, and differentiation mediated by STAT3 signaling pathways [59, 60]. Therefore, the role of IL-6 in mediating mouse MSCs in inflammation may be important and warrants further investigation.

## Conclusions

A large body of literature continues to focus on the therapeutic potential of MSCs in inflammatory and neoplastic disease processes, and the role of the NF-kB pathway continues to be studied. In the involvement of human MSCs in the pathogenesis of bowel cancer, it has been reported that β catenin in MSCs regulates NF-κB activity via members of the TNF receptor super family. De-regulated catenin activity in MSCs may contribute to the development of colorectal tumors, particularly in patients with inflammatory bowel disease [61]; given the effects of MSC differentiation *in vivo*, it is thought that human MSC osteogenesis is an important step in the development of vascular calcification. Studies report that the NF-κB pathway is central to complement receptor-mediated MSC osteogenesis and that modification of this may have a functional impact on vascular calcification [62]. This may be of particular importance in chronic inflammatory diseases, such as rheumatoid arthritis, which are associated with an increased risk of cardiovascular disease [63]. Furthermore, the NF-κB pathway has been implicated in enhanced migration and adhesion of human MSCs after exposure to TNFα [64].

These data all relate to the role of NF-κB signaling in relation to human MSCs. Therefore, we must consider the fact that murine studies of inflammatory conditions and MSCs reported in the literature may not predict what will be observed with human MSCs based on their responsiveness to NF-κB. It is clear that caution is required in the interpretation of *in vivo* studies of murine MSCs not only in CIA but in any condition where the inflammatory milieu may have unrecognized effects on MSC function and where the effect of cytokine networks on the cell may be related in part to the cells’ genetic background.

## Electronic supplementary material

Additional file 1: **Migration of FVB mesenchymal stem cells (MSCs)** ***in vitro.*** Migration of FVB MSCs was augmented by pre-stimulation with tumor necrosis factor-alpha (TNFα). This effect was not reversed by inhibition of the nuclear factor-kappa-B (NF-κB) pathway. (DOCX 20 KB)

## Abbreviations

7-AAD: 7-aminoactinomycin D
AdGFP: adenovirus expressing green fluorescent protein
CEM: complete expansion medium
CIA: collagen-induced arthritis
CIM: complete isolation medium
FBS: fetal bovine serum
GFP: green fluorescent protein
HS: horse serum
IFNγ: interferon-gamma
IL: interleukin
MAPK: mitogen-activated protein kinase
MHC: major histocompatibility complex
MOI: multiplicity of infection
MSC: mesenchymal stem cell
NF-κB: nuclear factor-kappa-B
P: passage
PBS: phosphate-buffered saline
P/S: penicillin/streptomycin
srIκB: super-repressor inhibitor of nuclear factor-kappa-B
STAT: signal transducer and activation of transcription
TNFα: tumor necrosis factor-alpha.

## References

[1] Barry FP, Murphy JM (2004). Mesenchymal stem cells: clinical applications and biological characterization. Int J Biochem Cell Biol.

[2] Pittenger MF, Mackay AM, Beck SC, Jaiswal RK, Douglas R, Mosca JD, Moorman MA, Simonetti DW, Craig S, Marshak DR (1999). Multilineage potential of adult human mesenchymal stem cells. Science.

[3] English K, Barry FP, Mahon BP (2008). Murine mesenchymal stem cells suppress dendritic cell migration, maturation and antigen presentation. Immunol Lett.

[4] Griffin MD, Ritter T, Mahon BP (2010). Immunological aspects of allogeneic mesenchymal stem cell therapies. Hum Gene Ther.

[5] Aggarwal S, Pittenger MF (2005). Human mesenchymal stem cells modulate allogeneic immune cell responses. Blood.

[6] Zappia E, Casazza S, Pedemonte E, Benvenuto F, Bonanni I, Gerdoni E, Giunti D, Ceravolo A, Cazzanti F, Frassoni F, Mancardi G, Uccelli A (2005). Mesenchymal stem cells ameliorate experimental autoimmune encephalomyelitis inducing T-cell anergy. Blood.

[7] Di Nicola M, Carlo-Stella C, Magni M, Milanesi M, Longoni PD, Matteucci P, Grisanti S, Gianni AM (2002). Human bone marrow stromal cells suppress T-lymphocyte proliferation induced by cellular or nonspecific mitogenic stimuli. Blood.

[8] Djouad F, Plence P, Bony C, Tropel P, Apparailly F, Sany J, Noël D, Jorgensen C (2003). Immunosuppressive effect of mesenchymal stem cells favors tumor growth in allogeneic animals. Blood.

[9] Corcione A, Benvenuto F, Ferretti E, Giunti D, Cappiello V, Cazzanti F, Risso M, Gualandi F, Mancardi GL, Pistoia V, Uccelli A (2006). Human mesenchymal stem cells modulate B-cell functions. Blood.

[10] Najar M, Raicevic G, Boufker HI, Fayyad Kazan H, De Bruyn C, Meuleman N, Bron D, Toungouz M, Lagneaux L (2010). Mesenchymal stromal cells use PGE2 to modulate activation and proliferation of lymphocyte subsets: combined comparison of adipose tissue, Wharton’s Jelly and bone marrow sources. Cell Immunol.

[11] Le Blanc K, Rasmusson I, Gotherstrom C, Seidel C, Sundberg B, Sundin M, Rosendahl K, Tammik C, Ringden O (2004). Mesenchymal stem cells inhibit the expression of CD25 (interleukin-2 receptor) and CD38 on phytohaemagglutinin-activated lymphocytes. Scand J Immunol.

[12] Potian JA, Aviv H, Ponzio NM, Harrison JS, Rameshwar P (2003). Veto-like activity of mesenchymal stem cells: functional discrimination between cellular responses to alloantigens and recall antigens. J Immunol.

[13] Majumdar MK, Keane-Moore M, Buyaner D, Hardy WB, Moorman MA, McIntosh KR, Mosca JD (2003). Characterization and functionality of cell surface molecules on human mesenchymal stem cells. J Biomed Sci.

[14] De Bari C, Dell’Accio F, Tylzanowski P, Luyten FP (2001). Multipotent mesenchymal stem cells from adult human synovial membrane. Arthritis Rheum.

[15] Augello A, Tasso R, Negrini SM, Cancedda R, Pennesi G (2007). Cell therapy using allogeneic bone marrow mesenchymal stem cells prevents tissue damage in collagen-induced arthritis. Arthritis Rheum.

[16] González MA, Gonzalez-Rey E, Rico L, Büscher D, Delgado M (2009). Treatment of experimental arthritis by inducing immune tolerance with human adipose-derived mesenchymal stem cells. Arthritis Rheum.

[17] Mao F, Xu WR, Qian H, Zhu W, Yan YM, Shao QX, Xu HX (2010). Immunosuppressive effects of mesenchymal stem cells in collagen-induced mouse arthritis. Inflamm Res.

[18] Liu Y, Mu R, Wang S, Long L, Liu X, Li R, Sun J, Guo J, Zhang X, Yu P, Li C, Liu X, Huang Z, Wang D, Li H, Gu Z, Liu B, Li Z (2010). Therapeutic potential of human umbilical cord mesenchymal stem cells in the treatment of rheumatoid arthritis. Arthritis Res Ther.

[19] Sullivan C, Murphy JM, Griffin MD, Porter RM, Evans CH, O’Flatharta C, Shaw G, Barry F (2012). Genetic mismatch affects the immunosuppressive properties of mesenchymal stem cells in vitro and their ability to influence the course of collagen-induced arthritis. Arthritis Res Ther.

[20] Djouad F, Fritz V, Apparailly F, Louis-Plence P, Bony C, Sany J, Jorgensen C, Noel D (2005). Reversal of the immunosuppressive properties of mesenchymal stem cells by tumor necrosis factor alpha in collagen-induced arthritis. Arthritis Rheum.

[21] Schurgers E, Kelchtermans H, Mitera T, Geboes L, Matthys P (2010). Discrepancy between the in vitro and in vivo effects of murine mesenchymal stem cells on T-cell proliferation and collagen-induced arthritis. Arthritis Res Ther.

[22] Chen B, Hu J, Liao L, Sun Z, Han Q, Song Z, Zhao RC (2010). Flk-1+ mesenchymal stem cells aggravate collagen-induced arthritis by up-regulating interleukin-6. Clin Exp Immunol.

[23] Choi JJ, Yoo SA, Park SJ, Kang YJ, Kim WU, Oh IH, Cho CS (2008). Mesenchymal stem cells overexpressing interleukin-10 attenuate collagen-induced arthritis in mice. Clin Exp Immunol.

[24] Papadopoulou A, Yiangou M, Athanasiou E, Zogas N, Kaloyannidis P, Batsis I, Fassas A, Anagnostopoulos A, Yannaki E (2012). Mesenchymal stem cells are conditionally therapeutic in preclinical models of rheumatoid arthritis. Ann Rheum Dis.

[25] Miettinen JA, Pietilä M, Salonen RJ, Ohlmeier S, Ylitalo K, Huikuri HV, Lehenkari P (2011). Tumor necrosis factor alpha promotes the expression of immunosuppressive proteins and enhances the cell growth in a human bone marrow-derived stem cell culture. Exp Cell Res.

[26] Zhang A, Wang Y, Ye Z, Xie H, Zhou L, Zheng S (2010). Mechanism of TNF-α-induced migration and hepatocyte growth factor production in human mesenchymal stem cells. J Cell Biochem.

[27] Wehling N, Palmer GD, Pilapil C, Liu F, Wells JW, Müller PE, Evans CH, Porter RM (2009). Interleukin-1beta and tumor necrosis factor alpha inhibit chondrogenesis by human mesenchymal stem cells through NF-kappaB-dependent pathways. Arthritis Rheum.

[28] Laschober GT, Brunauer R, Jamnig A, Singh S, Hafen U, Fehrer C, Kloss F, Gassner R, Lepperdinger G (2011). Age-specific changes of mesenchymal stem cells are paralleled by upregulation of CD106 expression as a response to an inflammatory environment. Rejuvenation Res.

[29] Jones E, Churchman SM, English A, Buch MH, Horner EA, Burgoyne CH, Reece R, Kinsey S, Emery P, McGonagle D, Ponchel F (2010). Mesenchymal stem cells in rheumatoid synovium: enumeration and functional assessment in relation to synovial inflammation level. Ann Rheum Dis.

[30] Zhao L, Huang J, Zhang H, Wang Y, Matesic LE, Takahata M, Awad H, Chen D, Xing L (2011). Tumor necrosis factor inhibits mesenchymal stem cell differentiation into osteoblasts via the ubiquitin E3 ligase Wwp1. Stem Cells.

[31] Lacey DC, Simmons PJ, Graves SE, Hamilton JA (2009). Proinflammatory cytokines inhibit osteogenic differentiation from stem cells: implications for bone repair during inflammation. Osteoarthritis Cartilage.

[32] Liu Y, Han ZP, Zhang SS, Jing YY, Bu XX, Wang CY, Sun K, Jiang GC, Zhao X, Li R, Gao L, Zhao QD, Wu MC, Wei LX (2011). Effects of inflammatory factors on mesenchymal stem cells and their role in the promotion of tumor angiogenesis in colon cancer. J Biol Chem.

[33] Lim H, Kim HP (2011). Matrix metalloproteinase-13 expression in IL-1β-treated chondrocytes by activation of the p38 MAPK/c-Fos/AP-1 and JAK/STAT pathways. Arch Pharm Res.

[34] Welf ES, Ahmed S, Johnson HE, Melvin AT, Haugh JM (2012). Migrating fibroblasts reorient directionality by a metastable, PI3K-dependent mechanism. J Cell Biol.

[35] He Z, Gao Y, Deng Y, Li W, Chen Y, Xing S, Zhao X, Ding J, Wang X (2012). Lipopolysaccharide induces lung fibroblast proliferation through Toll-like receptor 4 signaling and the phosphoinositide3-kinase-Akt pathway. PLoS One.

[36] Firestein GS, NF-kappa B (2004). Holy Grail for rheumatoid arthritis?. Arthritis Rheum.

[37] Cho HH, Shin KK, Kim YJ, Song JS, Kim JM, Bae YC, Kim CD, Jung JS (2010). NF-kappaB activation stimulates osteogenic differentiation of mesenchymal stem cells derived from human adipose tissue by increasing TAZ expression. J Cell Physiol.

[38] Böcker W, Docheva D, Prall WC, Egea V, Pappou E, Rossmann O, Popov C, Mutschler W, Ries C, Schieker M (2008). IKK-2 is required for TNF-alpha-induced invasion and proliferation of human mesenchymal stem cells. J Mol Med (Berl).

[39] Wang Y, Crisostomo PR, Wang M, Markel TA, Novotny NM, Meldrum DR (2008). TGF-alpha increases human mesenchymal stem cell-secreted VEGF by MEK- and PI3-K- but not JNK- or ERK-dependent mechanisms. Am J Physiol Regul Integr Comp Physiol.

[40] Ferrand J, Lehours P, Schmid-Alliana A, Mégraud F, Varon C (2011). Helicobacter pylori infection of gastrointestinal epithelial cells in vitro induces mesenchymal stem cell migration through an NF-κB-dependent pathway. PLoS One.

[41] Buhrmann C, Mobasheri A, Matis U, Shakibaei M (2010). Curcumin mediated suppression of nuclear factor-κB promotes chondrogenic differentiation of mesenchymal stem cells in a high-density co-culture microenvironment. Arthritis Res Ther.

[42] Jaiswal N, Haynesworth SE, Caplan AI, Bruder SP (1997). Osteogenic differentiation of purified, culture-expanded human mesenchymal stem cells in vitro. J Cell Biochem.

[43] Peister A, Mellad JA, Larson BL, Hall BM, Gibson LF, Prockop DJ (2004). Adult stem cells from bone marrow isolated from different strains of inbred mice vary in surface epitopes, rates of proliferation and differentiation potential. Blood.

[44] Eljaafari A, Tartelin ML, Aissaoui H, Chevrel G, Osta B, Lavocat F, Miossec P (2012). Bone marrow-derived and synovium-derived mesenchymal cells promote Th17 cell expansion and activation through caspase 1 activation: contribution to the chronicity of rheumatoid arthritis. Arthritis Rheum.

[45] Billiau A, Matthys P (2011). Collagen-induced arthritis and related animal models: how much of their pathogenesis is auto-immune, how much is auto-inflammatory?. Cytokine Growth Factor Rev.

[46] Doncarli A, Stasiuk LM, Fournier C, Abehsira-Amar O (1997). Conversion in vivo from an early dominant Th0/Th1 response to a Th2 phenotype during the development of collagen-induced arthritis. Eur J Immunol.

[47] Lubberts E, Joosten LA, Oppers B, van den Bersselaar L, Coenen-de Roo CJ, Kolls JK, Schwarzenberger P, van de Loo FA, van den Berg WB (2001). IL-1-independent role of IL-17 in synovial inflammation and joint destruction during collagen-induced arthritis. Immunol.

[48] Shahrara S, Pickens SR, Dorfleutner A, Pope RM (2009). IL-17 induces monocyte migration in rheumatoid arthritis. J Immunol.

[49] Kim JM, Jeong JG, Ho SH, Hahn W, Park EJ, Kim S, Yu SS, Lee YW, Kim S (2003). Protection against collagen-induced arthritis by intramuscular gene therapy with an expression plasmid for the interleukin-1 receptor antagonist. Gene Ther.

[50] Bocker W, Docheva D, Prall WC, Egea V, Pappou E, Rossmann O, Popov C, Mutschier W, Ries C, Schieker M (2007). IKK-2 is required for TNF-alpha-induced invasion and proliferation of human mesenchymal stem cells. J Mol Med (Berl).

[51] Shishodia S, Potdar P, Gairola CG, Aggarwal BB (2003). Curcumin (diferuloylmethane) down-regulates cigarette smoke-induced NF-kappaB activation through inhibition of IkappaBalpha kinase in human lung epithelial cells: correlation with suppression of COX-2, MMP-9 and cyclin D1. Carcinogenesis.

[52] Gu Q, Cai Y, Huang C, Shi Q, Yang H (2012). Curcumin increases rat mesenchymal stem cell osteoblast differentiation but inhibits adipocyte differentiation. Pharnacogn Mag.

[53] Sonowal H, Kumar A, Bhattacharyya J, Gogoi PK, Jaganathan BG (2013). Inhibition of actin polymerization decreases osteogeneic differentiation of mesenchymal stem cells through p38 MAPK pathway. J Biomed Sci.

[54] Yang K, Jiang Y, Han J, Gu J (2003). The binding of actin to p38 MAPK and inhibiting its kinase activity in vitro. Sci China C Life Sci.

[55] Xu DJ, Zhao YZ, Wang J, He JW, Weng YG, Luo JY (2012). Smads, p38 and ERK1/2 are involved in BMP9-induced osteogenic differentiation of C3H10T1/2 mesenchymal stem cells. BMB Rep.

[56] Watson JL, Greenshields A, Hill R, Hilchies A, Lee PW, Giacomantonio CA, Hoskin DW (2010). Curcumin-induced apoptosis in ovarian carcinoma cells is p53-independent and involves p38 mitogen-activated protein kinase activation and downregulation of Bcl-2 and survivin espression and Akt signalling. Mol Carcinog.

[57] Xia JM, Zhang J, Zhou WX, Liu XC, Han M (2013). Downregulation of p38 MAPK involved in inhibition of LDL-induced proliferation of mesangial cells and matrix by curcumin. J Huazhong Univ Sci Technol Med Sci.

[58] Cho JW, Lee KS, Kim CW (2007). Curcumin attenuates the espression of IL-1beta, IL-6 and TNF-alpha as well as cyclin E in TNF-alpha-treated HaCaT cells; NF-kappaB and MAPKs as potential upstream targets. Int J Mol Med.

[59] Lam SP, Luk JM, Man K, Ng KT, Cheung CK, Rose-John S, Lo CM (2010). Activation of interleukin-6-induced glycoprotein 130/signal transducer and activator of transcription 3 pathway in mesenchymal stem cells enhances hepatic differentiation, proliferation, and liver regeneration. Liver Transpl.

[60] Tondreau T, Meuleman N, Stamatopoulos B, De Bruyn C, Delforge A, Dejeneffe M, Martiat P, Bron D, Lagneaux L (2009). In vitro study of matrix metalloproteinase/tissue inhibitor of metalloproteinase production by mesenchymal stromal cells in response to inflammatory cytokines: the role of their migration in injured tissues. Cytotherapy.

[61] Schon S, Flierman I, Ofner A, Stahringer A, Holdt LM, Kolligs FT, Herbst A (2014). β-catenin regulates NF-κB activity via TNFRSF19 in colorectal cancer cells. Int J Cancer.

[62] Anaraki PK, Patecki M, Larmann J, Tkachuk S, Jurk K, Haller H, Theilmeier G, Dumler I (2014). Urokinase receptor mediates osteogenic differenatiation of mesenchymal stem cells and vascular calcigication via the complement C5a receptor. Stem Cells Dev.

[63] Avina-Zubieta JA, Choi HK, Sadatsafavi M, Etminan M, Esdaile JM, Lacaille D (2008). Risk of cardiovascular mortality in patients with rheumatoid arthritis: a meta-analysis of observational studies. Arthritis Rheum.

[64] Schmal H, Niemeyer P, Roesslein M, Hartl D, Loop T, Sudkamp NP, Stark GB, Mehlhorn AT (2007). Comparison of cellular functionality of human mesenchymal stromal cells and PBMC. Cytotherapy.

