# Supplementary material for: TNFα and IL-1β influence the differentiation and migration of murine MSCs independently of the NF-κB pathway
Source: Stem Cell Res Ther. 2014 Aug 27;5(4):104. doi: 10.1186/scrt492 (PMC4177434; doi:10.1186/scrt492)
Supplement: Supplementary file 1 — Additional file 1: Migration of FVB mesenchymal stem cells (MSCs) in vitro. Migration of FVB MSCs was augmented by pre-stimulation with tumor necrosis factor-alpha (TNFα). This effect was not reversed by inhibition of the nuclear factor-kappa-B (NF-κB) pathway. (DOCX 20 KB) [file 13287_2014_393_MOESM1_ESM.docx]

Supplementary Data

Migration of FVB MSCs was augmented by pre-stimulation with TNFα. This effect was not reversed by inhibition of the NF-κB pathway.
